# Does coenzyme Q10 improve semen quality and circulating testosterone level? a systematic review and meta-analysis of randomized controlled trials

**DOI:** 10.3389/fphar.2024.1497930

**Published:** 2025-01-03

**Authors:** Tunmise M. Akhigbe, Fabrael B. Fidelis, Adebayo O. Adekunle, Victory J. Ashonibare, Bolaji A. Akorede, Mansur S. Shuaibu, Suliat A. Hassan, Cecilia A. Adegbola, Precious J. Ashonibare, Opeyemi M. Oladapo, Adetomiwa E. Adeogun, Seun G. Bamidele, Precious A. Oyedokun, Mungala Mukolokota, Omotolani S. Kukoyi, Ayoola A. Oladipo, Olayinka E. Adelowo, Marvelous D. Akangbe, Jennifer R. Hughes, Albert M. Ricken, Martine Culty, Maria C. W. Avellar, Roland E. Akhigbe

**Affiliations:** ^1^ Department of Agronomy, Osun State University, Ejigbo campus, Osogbo, Osun, Nigeria; ^2^ Reproductive Biology and Toxicology Research Laboratory, Oasis of Grace Hospital, Osogbo, Osun, Nigeria; ^3^ Department of Biochemistry, Ahmadu Bello University, Zaria, Kaduna, Nigeria; ^4^ Department of Biological Sciences, Northern Arizona University, Flagstaff, AZ, United States; ^5^ Cardiovascular Regenerative Medicine & Tissue Engineering 3D Lab, Department of Cardiovascular Surgery and Research Group for Experimental Surgery, Medical Faculty, Heinrich Heine University, Düsseldorf, Germany; ^6^ Department of Biomedical Sciences, University of Wyoming, Laramie, United States; ^7^ Department of Biochemistry, Dokuz Eylul University, Izmir, Türkiye; ^8^ Department of Physiology, Ladoke Akintola University of Technology, Ogbomoso, Oyo, Nigeria; ^9^ Department of Physiology, Babcock University, Ilishan Remo, Ilishan Remo, Ogun, Nigeria; ^10^ Department of Physiological Sciences, Obafemi Awolowo University, Ife, Osun, Nigeria; ^11^ Department of Gastroenterology, Affiliated Hospital of Xuzhou Medical University, Xuzhou, Jiangsu, China; ^12^ Functional Microbiome Group, Uniklinik Aachen (Universitätsklinikum Aachen), Aachen, Germany; ^13^ Acrolytics LLC., Fort Collins, CO, United States; ^14^ Institute of Anatomy, Faculty of Medicine, University of Leipzig, Leipzig, Germany; ^15^ Department of Pharmacology and Pharmaceutical Sciences, Alfred E. Mann School of Pharmacy and Pharmaceutical Sciences, University of Southern California, Los Angeles, CA, United States; ^16^ Department of Pharmacology, Universidade Federal de São Paulo - Escola Paulista de Medicina, São Paulo, Brazil

**Keywords:** antioxidant, infertility treatment, male infertility, male reproduction, spermatogenesis, testes

## Abstract

**Background:**

Seminal oxidative stress has been shown to be a key factor in the development of male infertility. However, the benefits of infertility treatments with antioxidants such as coenzyme Q10 (CoQ10) remains controversial.

**Objectives:**

The aim of the present study was to assess the effects of CoQ10 supplementation on semen quality, i.e., semen volume, total sperm number, sperm concentration, total sperm motility, percentage of progressive sperm motility and sperm morphology. In addition, the effects of CoQ10 supplementation on circulating testosterone, luteinizing hormone (LH), follicle-stimulating hormone (FSH), and inhibin B levels were evaluated.

**Design:**

A systematic review and a meta-analysis of randomized controlled trials (RCTs) were performed to assess the effects of CoQ10 supplementation on semen quality and serum levels of male reproductive hormones.

**Methods:**

We conducted a strategic literature search in the Cochrane, EMBASE, PubMed/MEDLINE, Scopus, and Web of Science databases and collected only RCTs. The data in the collected RCTs were then meta-analyzed according to PRISMA guidelines.

**Results:**

Out of 2,144 collected studies, only eight were classified eligible. The studies included a total of 877 male subjects; 462 CoQ10-treated and 415 untreated/placebo-treated. We found significantly higher total sperm counts (SMD -13.38 [95% CI: −16.33, −10.43] *P*< 0.0001), total (SMD -7.26 [95% CI: −10.15, −4.36] *P<* 0.00001) and progressive motility (SMD -6.386 [95% CI: −10.04, −2.73] *P=* 0.0006), and normally formed sperm (SMD -1.96 [95% CI: −3.29, −0.62] *P=* 0.004) in CoQ10-treated male subjects compared with untreated/placebo-treated male subjects. Nonetheless, there was a significant inter-study heterogeneity in these studies. Moreover, significantly higher serum testosterone (SMD -0.59 [95% CI: −0.79, −0.40] *P<* 0.00001) and inhibin B levels (SMD -0.92 [95% CI: −1.47, −0.37] *P=* 0.001) were recorded in CoQ10-treated subjects compared to untreated/placebo-treated subjects. In addition, CoQ10 supplementation significantly lowered serum LH (SMD 1.77 [95% CI: 1.26, 2.28] *P<* 0.00001) and FSH concentrations (SMD 1.60 [95% CI: 1.38, 1.81] *P<* 0.00001). Interestingly, there was no significant inter-study heterogeneity in the hormonal studies. However, CoQ10 supplementation had no significant effect on semen volume (SMD 0.12 [95% CI: −0.13, 0.37] *P*= 0.34) and sperm concentration (SMD -6.69 [95% CI: −16.28, 2.90] *P=* 0.17).

**Conclusion:**

Our study shows that CoQ10 supplementation increases total sperm count, total and progressive sperm motility, and the proportion of normally formed sperm in association with higher serum testosterone and inhibin B levels. Our study therefore supports the view in the literature of a beneficial use of CoQ10 in male infertility treatment. However, further well-designed RCTs with sufficiently large numbers of subjects are required to reach a final conclusion.

## Introduction

Human fertility is progressively declining due to multiples new endogenous and exogenous factors, such as environmental contaminants, diet and behavior (Gallo, 2022; Rebuzzini et al., 2022). Worldwide, approximately one in six couples suffers from infertility (Hamed et al., 2023a; Akhigbe et al., 2022a; Ajayi and Akhigbe, 2020). and in roughly half of these cases, the cause is either partly or wholly attributable to the male partner (Hamed et al., 2023a; Akhigbe et al., 2022a; Ajayi and Akhigbe, 2020; Dissanayake et al., 2019; World Health Organization, 2010). In addition to newly emerging causes, (Ricken et al., 2024) the established causes of male infertility range from pre-testicular to testicular to post-testicular. The pretesticular causes include: Hypogonadotropic hypogonadism (due to congenital Kallmann’s syndrome or acquired causes such as trauma or tumors), chronic alcoholism, cigarette smoking, drugs and substance abuse. The testicular causes include: Congenital anomalies (e.g., Klinefelter’s syndrome), cryptorchidism, orchitis, testicular tumor, testicular torsion/detorsion, varicocele, radiotherapy and chemotherapy. The post-testicular causes include: Obstruction or absence of the vas deferens or ejaculatory duct, hypospadias and erectile dysfunction (Olooto, 2012).

Around 30%–40% of infertile men fall into the category of idiopathic male infertility (IMI), which includes physically, genetically and hormonally normal men who are unable to achieve fatherhood due to poor semen quality despite sufficient sexual intercourse (Salonia et al., 2021). Semen analysis is therefore of central importance in the diagnosis and management of male infertility (Akhigbe et al., 2022a; Dutta et al., 2021).

There is growing evidence that oxidative stress in seminal fluid is a key factor in poor semen quality, including in IMI (Akhigbe et al., 2022b; Oyedokun et al., 2023; Hamed et al., 2023b; Sharma and Agarwal, 1996; Takeshima et al., 2020). Seminal oxidative stress arises from an elevated presence of reactive oxygen species (ROS) within semen. The ROS predominantly originate from activated leukocytes (extrinsic ROS) and, to a lesser extent, from the sperms cells (intrinsic ROS) (Sharma and Agarwal, 1996). Increased formation of leukocytic ROS affects spermatogenesis in its early phases, resulting in morphologically flawed sperm with cytoplasmic remnants in their midpieces and abnormalities in their heads and tails. The presence of cytoplasmic residues in the midpiece elevates the proportion of cytoplasmic enzymes such as glucose-6-phosphate dehydrogenase in the sperm. The increased glucose-6-phosphate dehydrogenase activity leads to an accumulation of intracellular NADPH via the hexose monophosphate pathway, which in turn serves as an electron donor in the formation of intracellular ROS. Intrinsic ROS play vital roles during fertilization, including the induction of capacitation, enhancement of sperm motility and facilitation of acrosome reaction. (Aitken, 2017). However, excessive ROS concentrations within sperm initiate oxidations processes that may result in DNA fragmentation, a significant contributor of male infertility (Alvarez and Storey, 1995; Agarwal et al., 2017).

Given the role of oxidative stress in male infertility, there is growing interest in the potential benefits of antioxidants to enhancing semen quality. Antioxidants scavenge and neutralize free radicals, including the ROS found in semen, and improve semen quality (Ali et al., 2021). Antioxidants that have been evaluated include carnitine, carotenoids, cysteine, glutathione, lycopene, selenium, melatonin, vitamin C, vitamin E, and coenzyme Q10 (CoQ10), a lipophilic antioxydants (Barati et al., 2020; Abad et al., 2013; Smits et al., 2019; Lenzi et al., 2004; Gharagozloo et al., 2016; Arafa et al., 2020; Safarinejad et al., 2012). CoQ10 may be administered as the inactive form, ubiquinone, or as the active form, ubiquinol, which is better absorbed, and could lead to better bioavailability. Dietary sources of CoQ10 include organ meats, muscle meats, oily fish, legumes, nuts and seeds, and oils such as Soybean oil. However, these sources may not be sufficient to meet the demand of the body; hence, ingestion as supplements may be useful. Ingestion of CoQ10 (200 mg/day twice daily orally for half a year) results in detectable levels of CoQ10 in the semen and seminal plasma, and may increase sperm motility (Balercia et al., 2004). The *in vitro* study of Boonsimma et al. (2020) revealed that CoQ10 (50 μg/mL for 1 h) increased total sperm motility but not progressive sperm motility. Cakiroglu and his colleagues (Cakiroglu et al., 2014) reported that CoQ10 administration (100 mg twice daily for 6 months) improved sperm morphology and motility but not sperm concentration in subfertile men with astheno-teratozoospermia. Alahmar et al. (2021) showed that oral CoQ10 administration (200 mg/daily for 3 months) improved sperm concentration and motility in infertile men with idiopathic oligoasthenozoospermia. Festa et al. (2014) reported that CoQ10 supplementation (50 mg twice a day for 12 weeks) improves progressive sperm motility but not sperm morphology in infertile men.

Studies evaluating the effect of combined CoQ10 and one or two more antioxidants on semen quality show similar trends. Terai et al. (2020) reported that supplementation with a combination of antioxidants (L-carnitine, zinc, astaxanthin, coenzyme Q10, vitamin C, vitamin B12, and vitamin E) increased total sperm motility but did not significantly improve ejaculate volume, sperm count, or testosterone levels. Co-administration of CoQ10 and aspartic acid over 3 months was also observed to increase total and progressive sperm motility but not sperm concentration. (Tirabassi et al., 2015). However, Kopets et al. (2020) demonstrated that a combination of antioxidants (carnitine, arginine, zinc, vitamin E, glutathione, selenium, folic, CoQ10) improved ejaculate volume, total sperm motility, sperm count, and morphology.

Given the above information, it is evident that despite the possible benefits of CoQ10 supplementation on semen quality, the reported effects are controversial and the available data are contradictory, so further studies are needed. We therefore conducted a systematic review (SR) and meta-analysis (MA) to investigate the effects of CoQ10 supplementation on semen quality using data from identified RCTs. While conventional sperm parameters were considered primarily, male sex hormones levels, including testosterone, follicle-stimulating hormone (FSH), luteinizing hormone (LH), and inhibin B levels were considered secondarily.

## Materials and methods

### Study protocol and measured outcomes

The present MA was performed on previously published articles that documented the effect of CoQ10 supplementation on semen quality and male sex hormones. The Preferred Reporting Items for Systematic Reviews and Meta-Analysis (PRISMA) guide (Shamseer et al., 2015) was adopted to conduct this review. The impact of CoQ10 on ejaculate volume, sperm count, sperm concentration, total sperm motility, progressive sperm motility, and sperm morphology were considered as the primary outcomes, while the effect of CoQ10 on levels of circulating testosterone, LH, FSH, and inhibin were considered secondary outcomes.

### Eligibility criteria

This study was based on the focus question “Does Coenzyme Q10 supplementation improve semen quality and circulating testosterone level?“, which was designed in accordance with the Population, Intervention, Comparator, Outcome, and Study designs (PICO) framework (Mattos and Ruellas, 2015).

The inclusion eligibility criteria were:

i. Population: The studied population were male between 18 and 50 years old.
ii. Intervention: The RCTs evaluated the effect of CoQ10, when administered alone or in combination with other antioxidants, on semen quality and testosterone levels.
iii. Comparator: The study had an age-matched control group of subjects that were either placebo-treated or did not receive any treatment.
iv. Outcomes: The study reported conventional sperm parameters (ejaculate volume, sperm count, concentration, motility, and morphology) and male sex hormone levels (testosterone, FSH, LH, and inhibin) using mean values and standard deviation or any other values from which the mean values and standard deviations can be derived.
v. Study design: RCTs that answered the question “Does Coenzyme Q10 improve semen quality and circulating testosterone level?”.

The exclusion criteria were:

i. Population: The study did not contain any information on the age of the participants
ii. Exposure: Studies that used CoQ10 with other antioxidants but did not specify the antioxidants, or *in vitro* studies, or animal studies.
iii. Comparator: Studies without age-matched control.
iv. Outcomes: Studies that did not document the actual values of the variables of interest as mean and standard deviation or any other format from which the mean value and standard deviation can be calculated. In addition, studies that documented self-reported reproductive health outcome.
v. Study design: Cohort studies, case studies, review articles, commentaries, letters and editorials.

Other exclusion criteria included conference abstracts, preprint, degree thesis, and retracted papers. No language restriction was applied.

### Search strategies

All contributing authors (except JRH, AMR, MC, and MCWA) conducted a comprehensive literature search in the Cochrane, Google Scholar, Pubmed/MEDLINE, and Scopus databases by 30 January 2024, using these medical subject headings and Boolean operators: (“CoQ10”OR “Coenzyme Q10”OR “Q10” OR “antioxidant”) AND (“sperm” OR “sperm cell” OR “spermatozoa” OR “semen” OR “sperm count” OR “sperm concentration” OR “sperm motility” OR “semen volume” OR “ejaculate volume” OR “sperm morphology”) AND (“testosterone” OR “LH” OR “luteinizing hormone” OR “FSH” OR “follicle stimulating hormone” OR “inhibin” OR “hormone”). All eligible studies were collected. Also, citation-chasing technique was used to identify relevant papers. The abstracts of the retrieved articles were screened and the entire body of text were assessed by five investigators (MTA, FBF, VJA, CAA, and PJA) to confirm eligibility. Disagreements were resolved by a sixth investigator (REA).

### Assessment of the quality of the included studies and data extraction

The quality of evidence (QoE) of the included studies was evaluated using the Cochrane Risks-of-Bias tool for RCTs (RoB) (Supplementary Table S1) (Higgins et al., 2011) and the Grading of Recommendations Assessment, Development and Evaluation (GRADE) Working Group guidelines for the certainty of evidence. (Austin et al., 2014). QoE was evaluated by five investigators (TMA, FBF, VJA, CAA, and PJA), and disagreements were resolved by a sixth investigator (REA).

The relevant data were extracted from the eligible studies. Extracted information included; the last name of the first author, year of publication, the country where the study was executed, the number of study participants (sample size), the age of the subjects, the treatment administered, the duration of treatment, and the clinical outcomes measured. The data were extracted independently in triplicate (TMA, FBF, and VJA) for quality assurance, and a fourth investigator (REA) addressed discrepancies.

### Statistical analysis

MA was performed using Review Manager (RevMan) software (version 5.4.1). The data was analyzed using the random effect model if the *P*-value of the heterogeneity across the polled studies was less than 0.1 or I^2^ was greater than 50% (significant heterogeneity). However, when the *P*-value of the heterogeneity was ≥0.1 or I^2^ was greater ≤50% (low heterogeneity), the fixed effect model was used. (Huedo-Medina et al., 2006; Higgins et al., 2003). The publication bias was assessed by visual assessment of the funnel’s plot. Subgroup analyses were conducted to evaluated the impact of therapy duration (≤3 months treatment and >3 months treatment), and combination therapy (CoQ10 single therapy and CoQ10 combination therapy). Sensitivity analyses were conducted by eliminating the weightiest study, studies with at high risk, and studies with low confidence of evidence. *P* values less than 0.05 were considered statistically significant. Data on semen variables are presented as weighted mean difference (WMD) to preserve the original measurement units, facilitating interpretation while those on hormones are presented as standardized mean difference (SMD) since they were assayed by different methods in the eligible studies.

## Results

### Characteristics of the included studies

**FIGURE 1:**
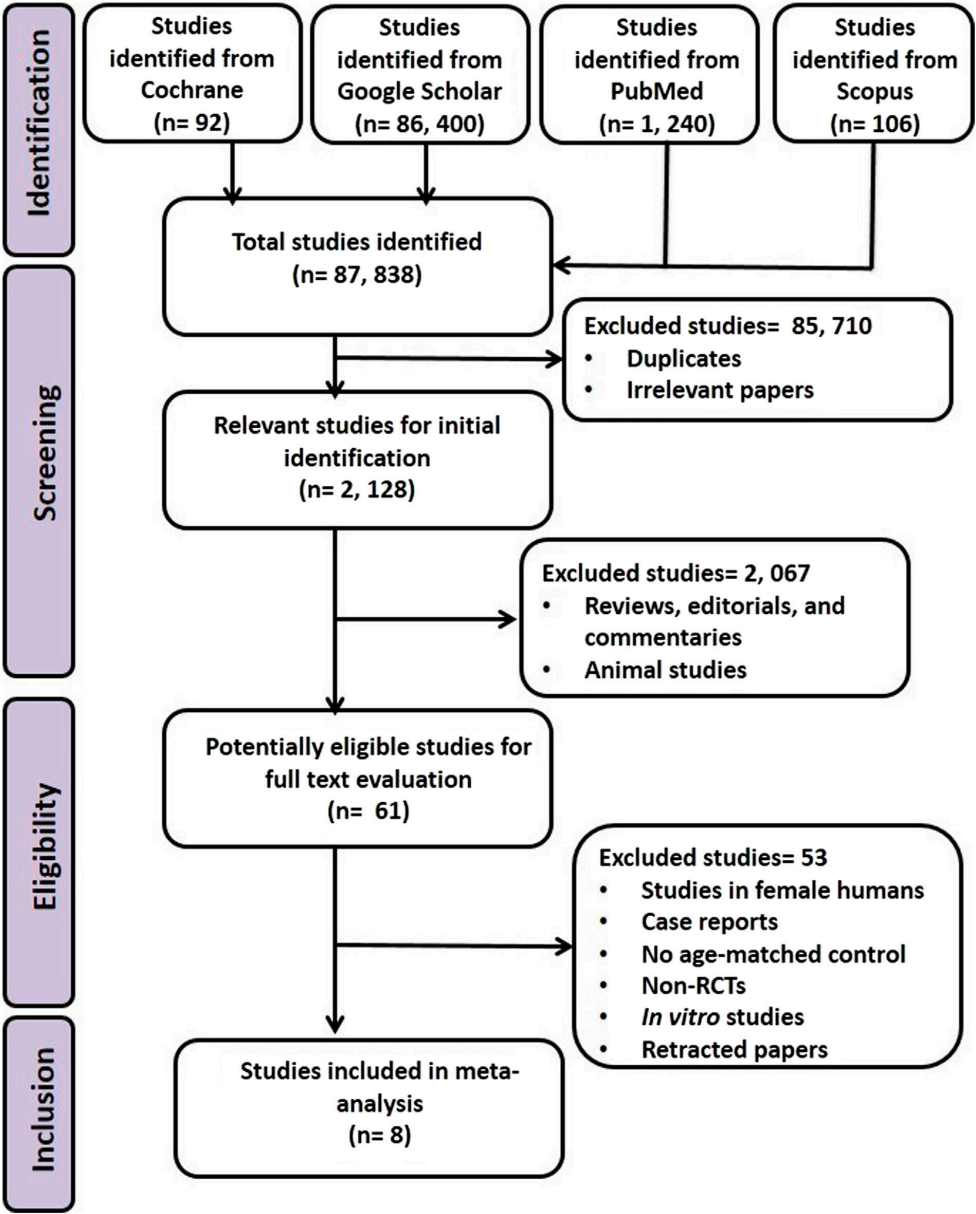
PRISMA flowchart for the identification, screening, and inclusion of eligible studies.

Using the pre-defined strategic protocol, 2,128 articles wereextracted from the initial 87,838 articles that were identified. After excluding studies that did not fulfill all of the inclusion criteria or met one or more of the exclusion criteria, eight articles were adjudged eligible (Figure 1).

All eligible studies included in the MA were RCTs and included at total of 877 male subjects (treated: 462, control: 415). The studies were from India (1), Iran (4), Italy (1), Sweden (1), and Ukraine (1). The age of the participants ranged from 18 to 50 years, and the duration of intervention was between 12 weeks and 26 weeks (3–6.5 months) (Table 1).

**TABLE 1:** Characteristics of the eligible studies included in the meta-analysis.

Reference	Study design	Country	Examined population	Age (years)	Intervention	Duration of intervention (months)	Outcomes/variables measured			Morphology technique
							Semen	Hormone	Others	
Balercia et al. (2009)	RCT	Italy	treated: 28 placebo: 30	27–39	CoQ10 (100 mg), lecithin, and medium chain glycerides vs. unspecified placebo twice daily	6 months	conc., total motility, forward motility, atypical morphology, COQ10, VSL; VCL, QH2, COQ10 sperm cells, QH2 sperm cells			
Gopinath et al. (2013) ^a^	RCT	India	Arm 1 (2 FDC): 46 Arm 2 (1 FDC, placebo): 43 Arm 3 (2 placebo): 36	24–45	CoQ10 (50 mg), L-carnitine (500 mg), lycopene (2.5 mg) and zinc (12.5 mg) vs. 1 tablet of FDC of antioxidants and one tablet of placebo or two tablets of matching placebo twice daily	3 and 6 months	amount total motility slow and rapid forward motility			
Nadjarzadeh et al., 2014	RCT	Iran	treated: 23 placebos: 24	25–40	CoQ10 (200 mg) vs. placebo daily	3 months	conc., total motility, forward motility, normal morphology			?
Nadjarzadeh et al., 2011	RCT	Iran	treated: 23 placebo: 24	25–46	CoQ10 (200 mg) vs. unspecified placebo (lactose) daily	12 weeks	volume, conc., total motility, forward motility, normal morphology	seminal plasma total antioxidant capacity, plasma malondialdehyde		?
Safarinejad et al. (2012)	RCT	? Iran	treated: 114 placebo: 114	25–44	CoQ10 (200 mg) of ubiquinol vs. unspecified placebo (10 mg) daily	26 weeks of treatment; 38 weeks (26 weeks of treatment +12 weeks off drug)	volume, amount, conc., total motility, normal morphology	testosterone, LH, FSH, prolactin, inhibin B	CAT, superoxide dismutase	Kruger
Safarinejad (2009)	RCT	? Iran	treated: 106 placebo: 106	21 and 42	CoQ10 (300 mg) of ubiquinol vs. unspecified placebo daily	26 weeks	volume amount, conc., total motility, morphology	testosterone, LH, FSH, PRL, inhibin B	IgA, IgG	Kruger
Stenqvist et al. (2018)	RCT	Sweden	treated: 37 placebo: 40	18–50	vitamins (vitamin C 30 mg, vitamin E 5 mg and vitamin B12 0.5 mg), antioxidants (l-carnitine 750 mg CoQ10 (10 mg) and folic acid (100 mg) and oligoelements (zinc 5 mg and selenium 25 mg) with maltodextrin, calcium carbonate, citric acid, steviol glycoside, flavours, beta-carotene and silicon dioxide vs. placebo (maltodextrin, calcium carbonate, citric acid, steviol glycoside, flavours, beta-carotene and silicon dioxide) daily	6 months	DFI, volume, amount, conc., total motility, forward motility, normal morphology			?
Kopets et al. (2020)	RCT	Ukraine	treated: 42 placebo: 41	21–50	(TDS) containing l-carnitine/acetyl-l-carnitine, l-arginine, glutathione, co-enzyme Q10, zinc, vitamin B9, vitamin B12, selenium, vs. unspecified placebo daily	6 months	conc., forward motility, normal morphology			Papanicolaou

^a^ Arm denotes the treatment groups as stated by the authors (Gopinath et al., 2013).

Arm 1 subjects received 2 tablets of FDC antioxidants twice daily, arm 2 subjects received 1 FDC tablet +1 placebo tablet twice daily and arm 3 subjects received 2 tablets of placebo twice daily. FDC, contains 50 mg of Co-Q10, 500 mg of L-carnitine, 12.5 mg of zinc 12.5, and 2.5 mg of lycopene 2.5 mg.

?: not stated

### QoE of the included studies

**FIGURE 2:**
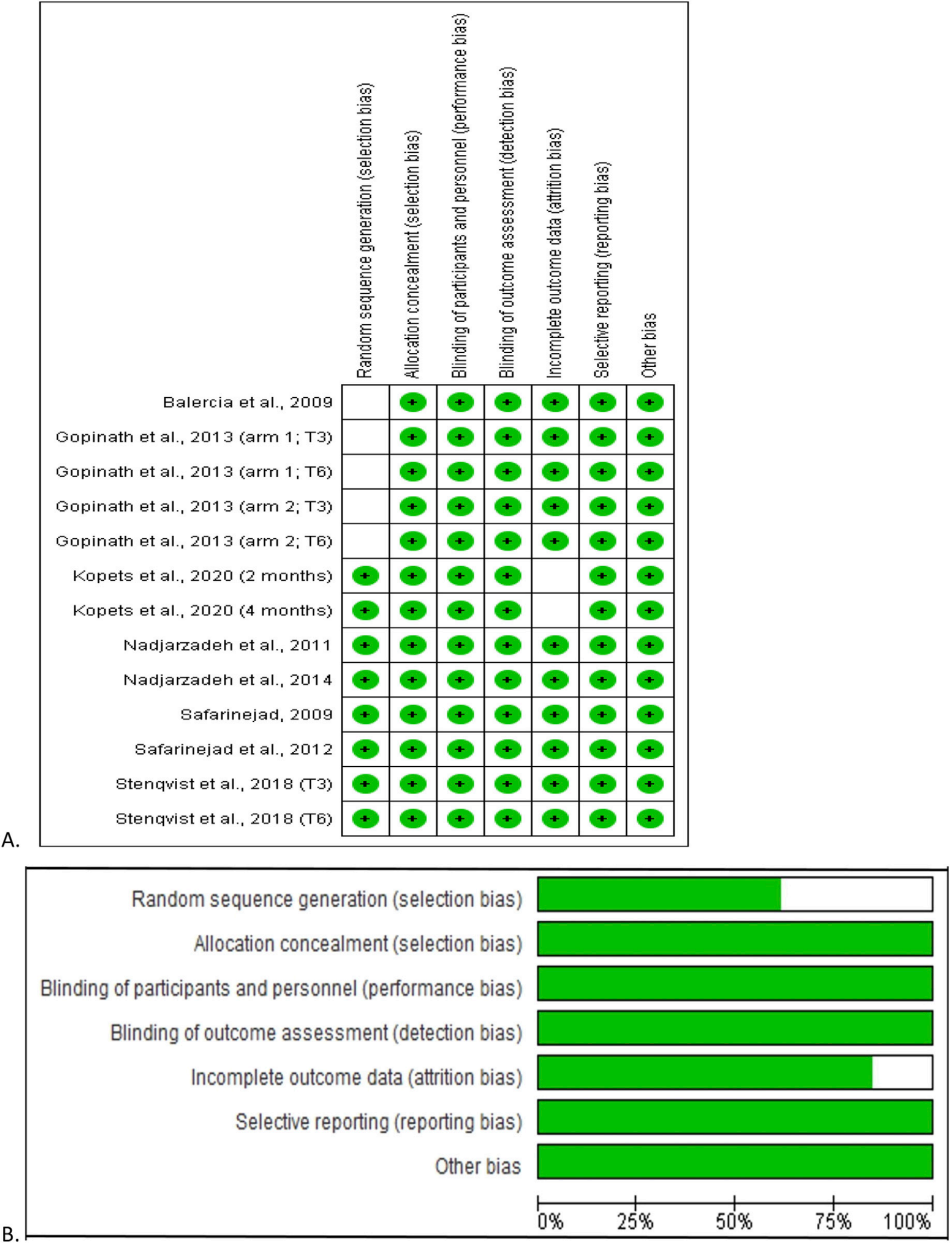
Risk of bias analysis of the included studies. The risk of bias summary showing each risk of bias item for each included study **(A)** and each risk of bias item presented as percentages across all included studies **(B)**. Green indicates the percentage probability that there is a low risk of bias.

With regard to the RoB, the studies by Balercia et al. (2009) and Gopinath et al. (2013) showed low RoB in all domains except for the domain “random sequence generation”, where the RoB was unclear. The study by Kopets et al. (2020) showed low RoB in all domains, with the exception of the “incomplete outcome data” domain, where the RoB was unclear. In the remaining studies (Safarinejad et al., 2012; Nadjarzadeh et al., 2014; Nadjarzadeh et al., 2011; Safarinejad, 2009; Stenqvist et al., 2018) a low RoB was measured in all domains. There was no study with high RoB in any domain (Figure 2A).

Overall, considering all eligible studies, the selection bias was 62.5% and 100% low risk in “random sequence generation” and “allocation concealment” respectively. In addition, 100% low risk was observed in performance bias, detection bias, reporting bias, and other bias, while about 82.5% of the studies had low risk for attrition bias (Figure 2B).

In two of the studies (Kopets et al., 2020; Nadjarzadeh et al., 2011), a moderate certainty of evidence was observed. However, other studies (Safarinejad et al., 2012; Balercia et al., 2009; Gopinath et al., 2013; Nadjarzadeh et al., 2014; Safarinejad, 2009; Stenqvist et al., 2018) showed a high certainty of evidence (Table 2).

**TABLE 2:** Certainty of evidence of the eligible studies.

Study	Initial confidences	Decreasing	Increasing	Final confidence
Balercia et al. (2009)	High	No	No	High
Gopinath et al. (2013)	High	No	No	High
Nadjarzadeh et al. (2014)	High	No	No	High
Nadjarzadeh et al. (2011)	High	Yes	No	Moderate
Safarinejad et al. (2012)	High	No	No	High
Safarinejad (2009)	High	No	No	High
Stenqvist et al. (2018)	High	No	No	High
Kopets et al. (2020)	High	Yes	No	Moderate

### Ejaculate volume

**FIGURE 3:**
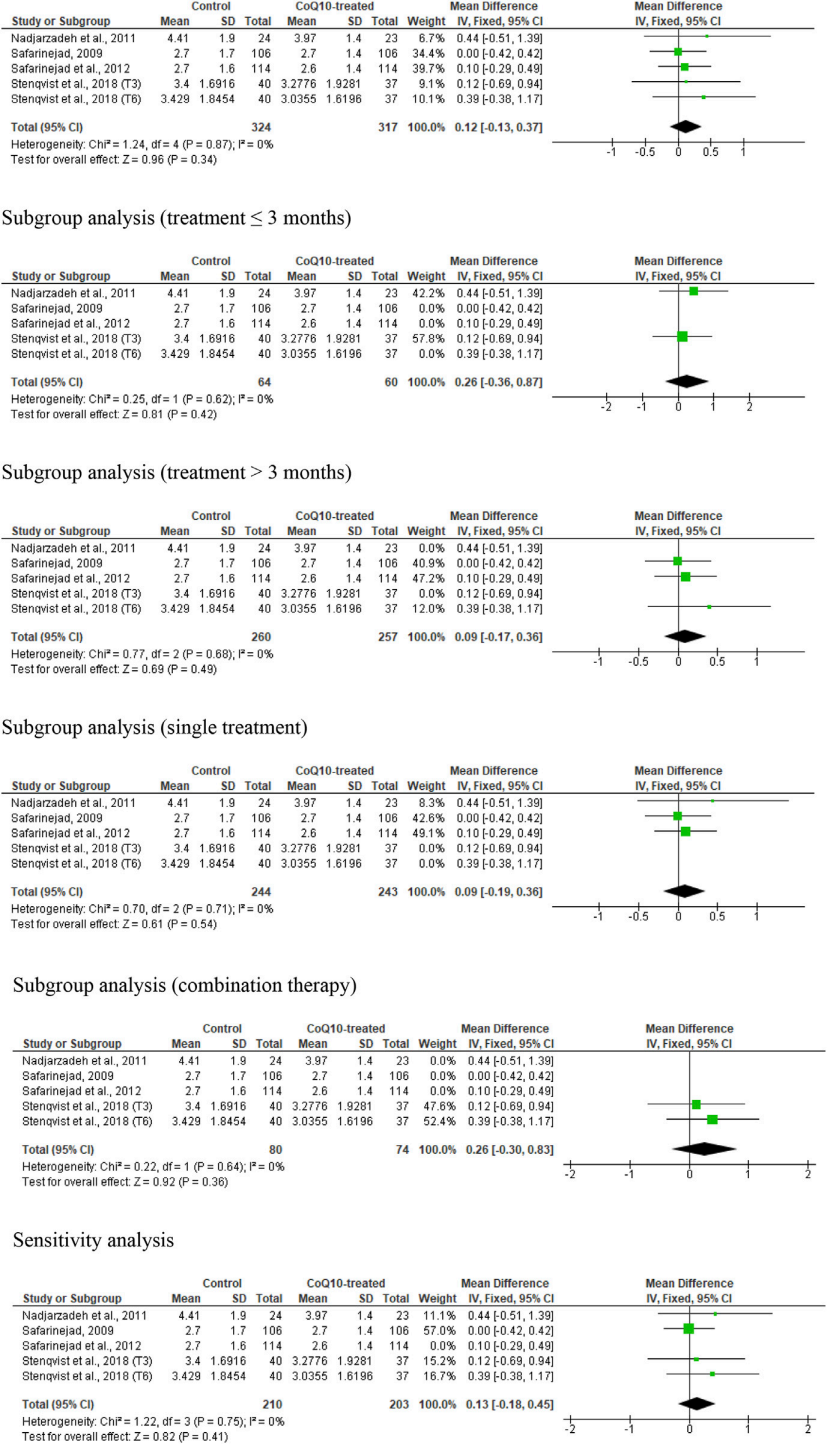
CoQ10 supplementation does not change ejaculate volume (mL). In the unrestricted, sugbroup and sensitivity analyses, there were no changes in ejaculate volume after CoQ10 supplementation and this was consistent across studies. The green boxes represent the mean effects of the included studies, while the diamond-shaped black boxes are the global mean effects of all studies. The boxes on the right show that the variable is higher in the control.

Five studies from four RCTs (Safarinejad et al., 2012; Nadjarzadeh et al., 2011; Safarinejad, 2009; Stenqvist et al., 2018) assessed ejaculate volume in association with CoQ10 supplementation, including 641 subjects (324 in the untreated or placebo-treated control group and 317 in the CoQ10-treated group). There was no substantial change in ejaculate volume (WMD 0.12 [95% CI: −0.13, 0.37] *P*= 0.34) and there was no significant inter-study heterogeneity (I^2^ = 0%; *X* ^2^ *P =* 0.87) (Figure 3). There was a significant publication bias (Supplementary Figure S1A). The subgroup analyses of studies that evaluated the effect of CoQ10 use for ≤3 months (WMD 0.26 [95% CI: −0.36, 0.87] *P*= 0.42), >3 months (WMD 0.09 [95% CI: −0.17, 0.36] *P*= 0.34), as a single therapy (WMD 0.09 [95% CI: −0.19, 0.36] *P*= 0.54), and as a combined therapy (WMD 0.26 [95% CI: −0.30, 0.83] *P*= 0.36) also showed that CoQ10 did not significantly alter ejaculate volume. More so, the sensitivity analysis performed after the exclusion of weightiest study revealed that CoQ10 did not alter ejaculate volume (WMD 0.13 [95% CI: −0.18, 0.45] *P*= 0.41) and there was no significant inter-study heterogeneity (I^2^ = 0%; *X* ^2^ *P =* 0.75) (Figure 3).

Overall, based on the statistical information, it appeared that CoQ10 supplementation did not significantly alter ejaculate volume in male subjects; there was no significant heterogeneity across studies. However, there was evidence of publication bias.

### Sperm count

**FIGURE 4:**
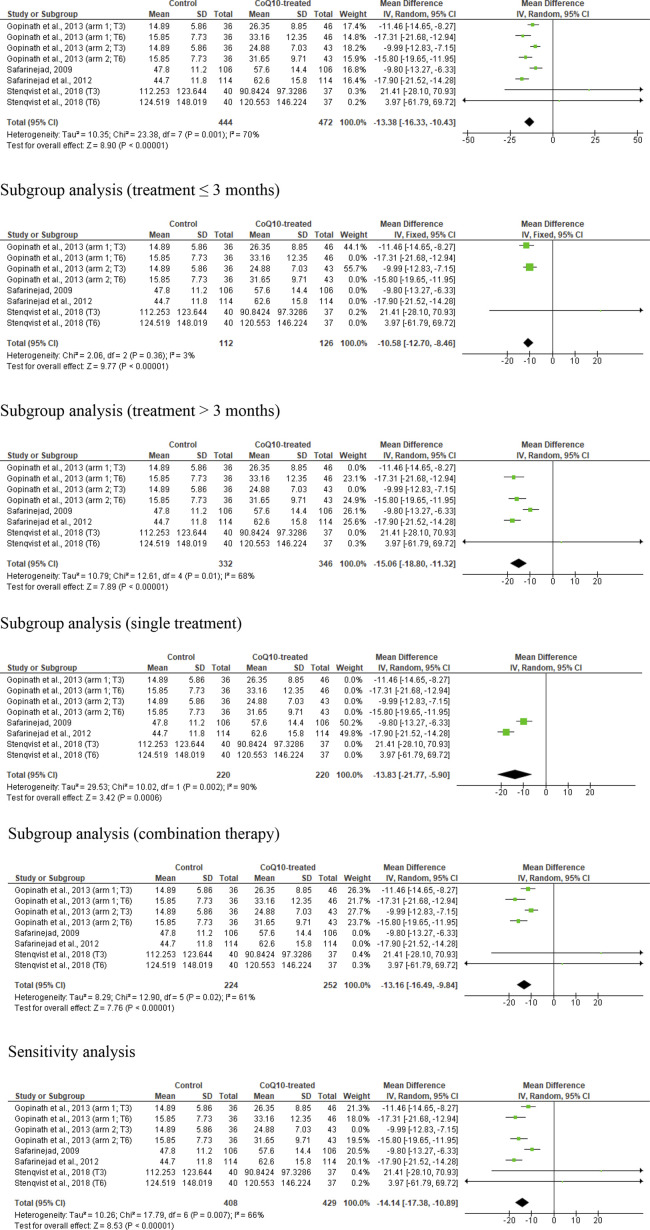
CoQ10 supplementation had consistent effects on total sperm count (million). The unrestricted analysis of all studies resulted in significantly higher total sperm count in CoQ10-treated individuals. Also, the subgroup analyses for different durations and combinations of treatment, and the sensitivity analysis revealed that CoQ10 treatment significantly increased sperm count. The green boxes represent the mean effects of the included studies, while the diamond-shaped black boxes are the global mean effects of all studies. The boxes on the left side show that the variable is higher in CoQ10-treated groups, while the boxes on the right show that the variable is higher in the control.

Eight studies from four articles were considered eligible in the evaluation of sperm count. (Safarinejad et al., 2012; Gopinath et al., 2013; Safarinejad, 2009; Stenqvist et al., 2018). This included 444 untreated or placebo-treated control subjects and 472 CoQ10-treated subjects. The analysis demonstrated a significant increase in sperm count in CoQ10-treated subjects (WMD -13.38 [95% CI: −16.33, −10.43] *P*< 0.0001) when compared with the control but there was a significant inter-study heterogeneity (I^2^ = 70%; *X* ^2^ *p =* 0.001) (Figure 4). There was a significant publication bias (Supplementary Figure S1B). The subgroup analyses of studies that assessed the impact of CoQ10 therapy for ≤3 months (WMD -10.58 [95% CI: −12.70, −8.46] *P*< 0.00001) and >3 months (WMD -15.06 [95% CI: −18.80, −11.32] *P*< 0.00001) revealed that CoQ10 significantly increased sperm count when compared to the control. Also, the subgroup analyses for the impact of CoQ10 as a single therapy (WMD -13.83 [95% CI: −21.77, −5.90] *P*= 0.0006) and as combined therapy (WMD -13.16 [95% CI: −16.49, −9.84] *P*< 0.00001) showed that CoQ10 significantly increased sperm count. Furthermore, the sensitivity analysis of the eligible studies showed that CoQ10 supplementation significantly increased sperm count (WMD -14.14 [95% CI: −17.38, −10.89] *P*< 0.00001) but there was also a significant inter-study heterogeneity (I^2^ = 66%; *X* ^2^ *P =* 0.007) (Figure 4).

In summary, the unrestricted statistical analysis as well as the subgroup and sensitivity analyses suggested a significant increase in sperm count with CoQ10 supplementation.

### Sperm concentration

**FIGURE 5:**
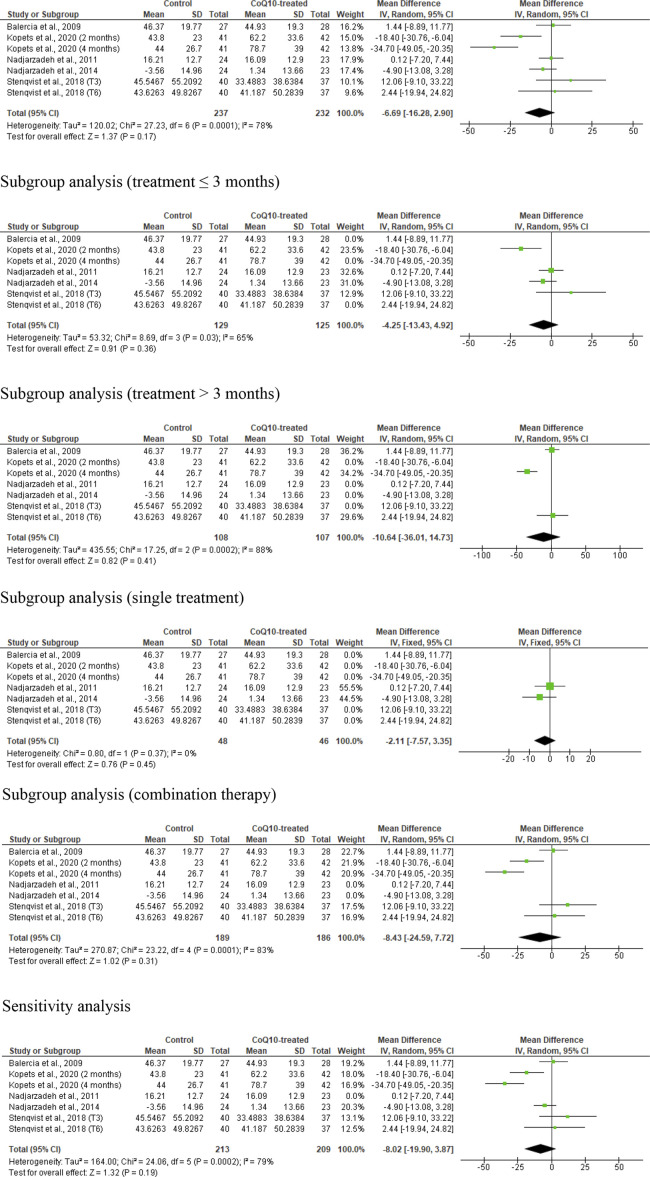
CoQ10 supplementation had no effect sperm concentration (million/mL). Sperm concentration did not change in the unrestricted, and subgroup and sensitivity analyses. There were significant differences between studies, indicated by the significant heterogenity of both analysis. The green boxes represent the mean effects of the included studies, while the diamond-shaped black boxes are the global mean effects of all studies. The boxes on the left side show that the variable is higher in CoQ10-treated groups, while the boxes on the right show that the variable is higher in the control.

Seven studies from five RCTs were examined in the analysis of sperm concentration (Kopets et al., 2020; Balercia et al., 2009; Nadjarzadeh et al., 2014; Nadjarzadeh et al., 2011; Stenqvist et al., 2018), including 237 untreated or placebo-treated control subjects and 232 CoQ10-treated subjects. The analysis showed no significant change in sperm concentration (WMD -6.69 [95% CI: −16.28, 2.90] *P=* 0.17) but there was a significant inter-study heterogeneity (I^2^ = 78%; *X* ^2^ *P=* 0.0001) (Figure 5). There was a significant publication bias (Supplementary Figure S1C). The subgroup analyses for CoQ10 use for ≤3 months (WMD -4.25 [95% CI: −13.43, 4.92] *P*= 0.36), >3 months (WMD 10.64 [95% CI: −36.01, 14.73] *P*= 0.41), as a single therapy (WMD -2.11 [95% CI: −7.57, 3.35] *P*= 0.45), and as a combined therapy (WMD -8.43 [95% CI: −24.59, 7.72] *P*= 0.31) also showed that CoQ10 did not significantly change sperm concentration. More so, the sensitivity analysis of the eligible studies also revealed that CoQ10 supplementation did not significantly increase sperm concentration (WMD -8.02 [95% CI: −19.90, 3.87] *P*= 0.19), but there was also a significant inter-study heterogeneity (I^2^ = 79%; *X* ^2^ *P=* 0.0002) (Figure 5).

Over all, the analysis suggested that CoQ10 supplementation did not significantly affect sperm concentration, with variability and publication bias evident across the studies.

### Sperm total motility

**FIGURE 6:**
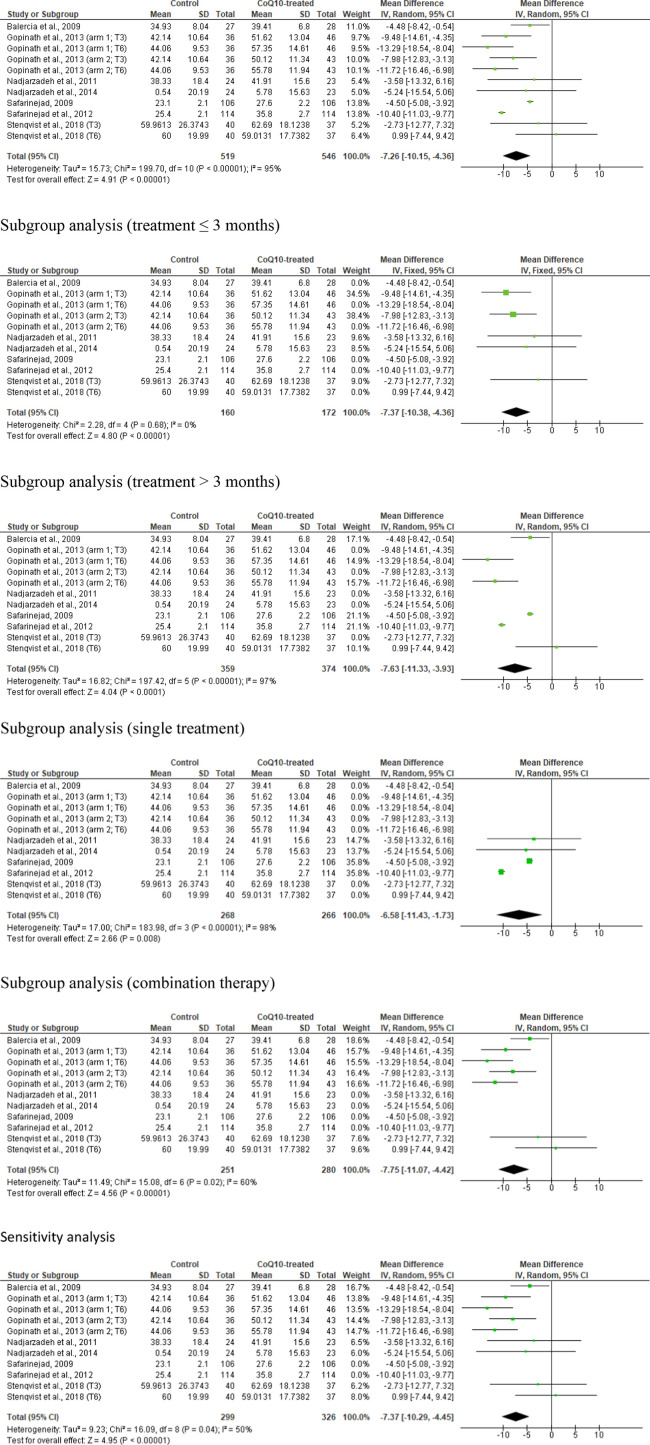
CoQ10 supplementation had a consistent effect on total sperm motility (%). The unrestricted analysis of all studies showed a significant increase in total sperm motility in CoQ10-treated group compared to the control. This significant difference psersisted with the various subgroup analyses and sensitivity analysis. The green boxes represent the mean effects of the included studies, while the diamond-shaped black boxes are the global mean effects of all studies. The boxes on the left side show that the variable is higher in CoQ10-treated groups.

Eleven studies from seven articles were examined in the meta-analysis of total sperm motility (Safarinejad et al., 2012; Balercia et al., 2009; Gopinath et al., 2013; Nadjarzadeh et al., 2014; Nadjarzadeh et al., 2011; Safarinejad, 2009; Stenqvist et al., 2018). These studies included 519 untreated or placebo-treated control subjects and 546 CoQ10-treated subjects. There was a noticeable increase in the total sperm motility in the CoQ10-treated subjects when compared with the untreated males (WMD −7.26 [95% CI: −10.15, −4.36] *P<* 0.00001) but there was a significant inter-study heterogeneity (I^2^ = 95%; *X* ^2^ *P<* 0.00001) (Figure 6). There was a significant publication bias (Supplementary Figure S1D). The subgroup analyses for CoQ10 use for ≤3 months (WMD -7.37 [95% CI: −10.38, −4.36] *P*< 0.00001) and >3 months (WMD -7.63 [95% CI: −11.33, −3.93] *P*< 0.0001) showed that CoQ10 use significantly increased sperm total motility when compared with the control. The subgroup analyses for CoQ10 as a single therapy (WMD -6.58 [95% CI: −11.43, −1.73] *P*= 0.008) and a combined therapy (SMD -7.75 [95% CI: −11.07, −4.42] *P*< 0.00001) also showed that CoQ10 significantly increased sperm total motility. Interestingly, the sensitivity analysis also showed that CoQ10 supplementation significantly increased sperm total motility (WMD -7.37 [95% CI: −10.29, −4.45] *P*< 0.00001) and there was no significant inter-study heterogeneity (I^2^ = 98%; *X* ^2^ *P<* 0.00001) (Figure 6).

In summary, the meta-analysis revealed a noticeable increase in sperm total motility in males treated with CoQ10 compared to untreated subjects. However, significant inter-study heterogeneity and publication bias were noted. Also, sensitivity analysis showed a significant positive impact of CoQ10 supplementation on sperm total motility and there was no considerable inter-study heterogeneity.

### Sperm progressive motility

**FIGURE 7:**
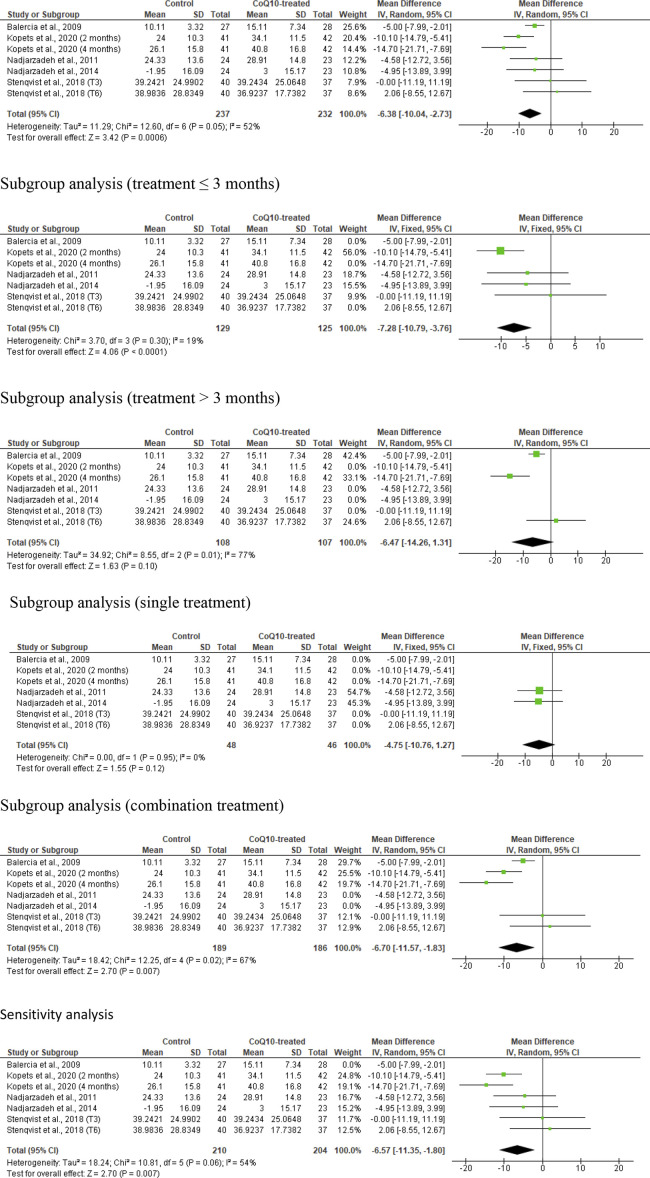
CoQ10 supplementation significantly increased sperm progressive motility (%). The unrestricted analysis, subgroup analyses for ≤3 months of treatment and combined therapy, and sensitivity analysis demonstrated a significant increase in sperm progressive motility in CoQ10-treated subjects when comapred to the control. However, subgroup analysis for >3 months of treatment and single therapy revealed an insignificant increase in the percentage of sperm progressive motility after CoQ10 supplementation. The green boxes represent the mean effects of the included studies, while the diamond-shaped black boxes are the global mean effects of all studies. The boxes on the left side show that the variable is higher in CoQ10-treated groups, while the boxes on the right show that the variable is higher in the control.

Seven studies from five articles were included in the meta-analysis of the effect of CoQ10 supplementation on sperm progressive motility, (Balercia et al., 2009; Gopinath et al., 2013; Nadjarzadeh et al., 2014; Nadjarzadeh et al., 2011; Stenqvist et al., 2018) so that 237 untreated or placebo-treated control subjects and 232 CoQ-treated subjects were compared. There was a substantial improvement in the progressive sperm motility in CoQ10-treated subjects in comparison with the control (WMD -6.386 [95% CI: −10.04, −2.73] *P=* 0.0006) but there was a notable inter-study heterogeneity (I^2^ = 52%; *X* ^2^ *P=* 0.05) (Figure 7). There was also a significant publication bias (Supplementary Figure S1E). Subgroup analyses revealed that there was a significant increase in sperm progressive motility after CoQ10 use for ≤3 months (WMD -7.28 [95% CI: −10.79, −3.76] *P*< 0.0001) but not for >3 months (WMD -14.26 [95% CI: −14.26, 1.31] *P*= 0.10). Also, CoQ10 as a single therapy did not significant alter sperm progressive motility when compared with the control (WMD -4.75 [95% CI: −10.76, 1.27] *P*= 0.12), but it did as a combination therapy (WMD -6.70 [95% CI: −11.57, −1.83] *P*= 0.007). Additionally, the sensitivity analysis showed that CoQ10 supplementation significantly increased sperm progressive motility (WMD -6.57 [95% CI: −11.35, −1.80] *P*= 0.007) but there was significant inter-study heterogeneity (I^2^ = 54%; *X* ^2^ *P=* 0.06) (Figure 7).

In summary, the meta-analysis demonstrated significant improvement in progressive sperm motility with CoQ10 supplementation, albeit with notable heterogeneity among the studies. Although there were mixed findings with subgroup analyses showing that combined therapy, but not prolonged treatment, influenced the outcome, the sensitivity analysis reaffirmed the positive effect of CoQ10 supplementation on sperm progressive motility, but with a considerable inter-study heterogeneity.

### Sperm normal morphology

**FIGURE 8:**
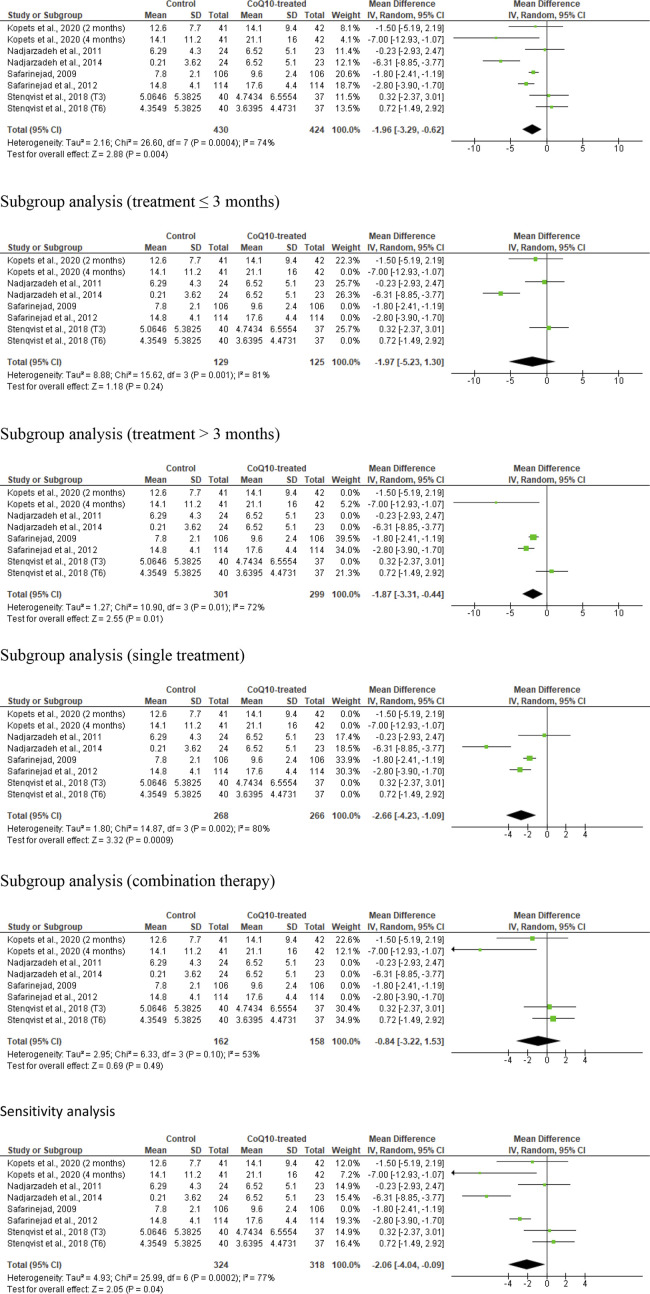
CoQ10 supplementation significantly increased normal sperm morphology. In the unresssicted analysis, there was a significant increase in sperm morphology after CoQ10 supplementation, however these findings were inconsistent between studies. Subgroup analyses of treatments >3 months and single therapy also showed significant increase of normal sperm morphology in CoQ10-treated group compared with the control, but subgroup analyses of treatments ≤3 months and combined therapy demonstrated that CoQ10 did not alter normal sperm morphology. Nonetheless, the sensitivity analysis showed a significant increase in sperm morphology in CoQ10-treated group compared with the control. The green boxes represent the mean effects of the included studies, while the diamond-shaped black boxes are the global mean effects of all studies. The boxes on the left side show that the variable is higher in CoQ10-treated groups, while the boxes on the right show that the variable is higher in the control.

Eight studies from six articles, (Safarinejad et al., 2012; Mattos and Ruellas, 2015; Nadjarzadeh et al., 2014; Nadjarzadeh et al., 2011; Safarinejad, 2009; Stenqvist et al., 2018), comparing 430 untreated or placebo-treated control subjects and 424 CoQ-treated subjects, were eligible for the evaluation of the impact of CoQ10 on sperm morphology. There was a notable improvement in the normal sperm morphology in CoQ10-treated subjects compared to control subjects (WMD -1.96 [95% CI: −3.29, −0.62] *P=* 0.004) but there was a significant inter-study heterogeneity (I^2^ = 74%; *X* ^2^ *P=* 0.0004) (Figure 8). There was significant publication bias (Supplementary Figure S1F). The subgroup analysis of studies that evaluated the effect of CoQ10 use for >3 months (WMD -1.87 [95% CI: −3.31, −0.44] *P*= 0.01), but not ≤3 months (WMD -1.97 [95% CI: −5.23, 1.30] *P*= 0.24), demonstrated that CoQ10 significantly increased the percentage of sperm normal morphology. Also, the use of CoQ10 as a single therapy (WMD −2.66 [95% CI: −4.23, −1.09] *P*= 0.0009), but not as a combined therapy (WMD -0.84 [95% CI: −3.22, 1.53] *P*= 0.49), significantly improved sperm normal morphology. More so, sensitivity analysis revealed that CoQ10 supplementation markedly increased sperm normal morphology (WMD −2.06 [95% CI: −4.04, −0.09] *P*= 0.04) but there was a significant inter-study heterogeneity (I^2^ = 77%; *X* ^2^ *P=* 0.0002) (Figure 8).

In summary, the meta-analysis demonstrated that male subjects who received CoQ10 supplementation showed a significantly higher proportion of normally formed sperm compared to the control group. Although there were mixed findings with subgroup analyses showing that the prolonged use of CoQ10 (>3 months) and the use as a single therapy, but not the use for ≤3 months and as a combined therapy, significantly improved sperm normal morphology; the sensitivity analysis revealed a substantial increase in normally formed sperm with CoQ10 supplementation, but again there was considerable heterogeneity between the studies.

### Circulating male sex hormones

**FIGURE 9:**
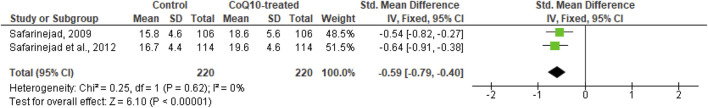
CoQ10 supplementation significantly increased serum testosterone. Two studies measured circulating testosterone levels in men after 26 weeks of treatment with CoQ10 or placebo. CoQ10 treatment resulted in significantly increased testosterone levels. These findings were consistent across studies and this data cooberates the decreased levels of serum LH and FSH from the same studies. The green boxes represent the mean effects of the included studies, while the diamond-shaped black boxes are the global mean effects of all studies. The boxes on the left side show that the variable is higher in CoQ10-treated groups.

Just two studies were included in the analysis of the effect of CoQ10 supplementation on serum testosterone, LH, FSH, and Inhibin B levels (Safarinejad et al., 2012; Safarinejad, 2009). These studies included 220 untreated males and 220 CoQ10-treated males. It was observed that CoQ10 significantly increased serum testosterone levels (SMD -0.59 [95% CI: −0.79, −0.40] *P<* 0.00001) and there was no significant inter-study heterogeneity (I^2^ = 0%; *X* ^2^ *P=* 0.62). There was no significant publication bias (Supplementary Figure S1G). A sensitivity analysis was not performed due to limited data on the effect of CoQ10 on serum testosterone levels (Figure 9).

**FIGURE 10:**
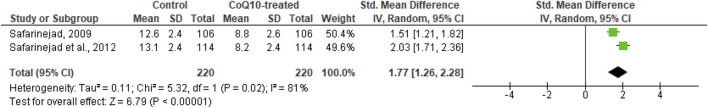
CoQ10 supplementation significantly lowered serum LH. Two studies measured circulating LH levels in men after 26 weeks of treatment with CoQ10 or placebo. CoQ10 treatment resulted in significantly lowered LH levels. These findings were consistent across studies and this data cooberates the increased levels of serum testosterone from the same studies. The green boxes represent the mean effects of the included studies, while the diamond-shaped black boxes are the global mean effects of all studies. The on the right show that the variable is higher in the control.

In addition, CoQ10 significantly reduced serum LH levels (SMD 1.77 [95% CI: 1.26, 2.28] *P<* 0.00001) but there was a significant inter-study heterogeneity (I^2^ = 81%; *X* ^2^ *P=* 0.02). There was no significant publication bias (Supplementary Figure S1H). Sensitivity analysis was not carried out due to limited data on the effect of CoQ10 on serum LH levels (Figure 10).

**FIGURE 11:**
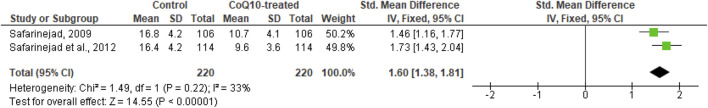
CoQ10 supplementation significantly lowered serum FSH. Two studies measured circulating FSH level in men after 26 weeks of treatment with CoQ10 or placebo. CoQ10 treatment resulted in significantly lowered FSH levels. These findings were consistent across studies and this data cooberates the increased levels of serum testosterone from the same studies. The green boxes represent the mean effects of the included studies, while the diamond-shaped black boxes are the global mean effects of all studies. The boxes on the right show that the variable is higher in the control.

Furthermore, CoQ10 significantly reduced serum FSH levels (SMD 1.60 [95% CI: 1.38, 1.81] *P<* 0.00001) and there was no significant inter-study heterogeneity (I^2^ = 33%; *X* ^2^ *P=* 0.22). We found no publication bias of significance (Supplementary Figure S1I). Sensitivity analysis was not conducted due to limited data on the effect of CoQ10 on serum FSH levels (Figure 11).

**FIGURE 12:**
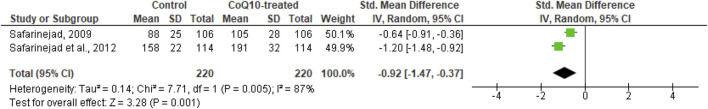
CoQ10 supplementation significantly increased serum Inhibin B. Two studies measured circulating Inhibin B levels in men after 26 weeks of treatment with CoQ10 or placebo. CoQ10 treatmetn resulted in significantly higher Inhibin B levels. These findings were consistent across studies and this data cooberates the increased levels of serum testosterone from the same studies. The green boxes represent the mean effects of the included studies, while the diamond-shaped black boxes are the global mean effects of all studies. The boxes on the left side show that the variable is higher in CoQ10-treated groups.

Moreover, CoQ10 significantly increased serum inhibin B levels (SMD -0.92 [95% CI: −1.47, −0.37] *P=* 0.001) but there was a significant inter-study heterogeneity (I^2^ = 87%; *X* ^2^ *P=* 0.005). There was no significant publication bias (Supplementary Figure S1J). Sensitivity analysis was not performed due to limited data on the effect of CoQ10 on serum inhibin B levels (Figure 12).

In summary the results indicates that CoQ10 supplementation significantly increase serum testosterone levels while reducing serum LH and FSH levels compared to untreated subjects. Moreover, CoQ10 supplementation seems to correlate with a significant increase in serum inhibin B levels. However, noteworthy inter-study heterogeneity is observed in some analyses, and there are insufficient data for sensitivity analysis across all parameters. Nevertheless, no significant publication bias is detected in any of the analyses.

## Discussion

Semen analysis remains pivotal to the treatment of male infertility. It typically serves as the initial diagnostic investigation and it is a valuable tool for tracking a patient’s response to treatment. While the ultimate goal of managing male infertility is achieving a clinical pregnancy or live birth, conventional semen analysis remains a useful tool in the management of male infertility. In the present meta-analysis, there was a convincing evidence of a significant improvement in sperm count, progressive motility, normal morphology, and circulating testosterone levels following CoQ10 supplementation. Although the ejaculate volume and sperm concentration were not significantly improved, the data presented in this study are convincing and provide further evidence of the therapeutic potentials of CoQ10 supplementation in the management of male infertility.

Our results, indicating that CoQ10 supplementation improves sperm count, progressive motility, and normal morphology are consistent with previous studies (Boonsimma et al., 2020; Shams-Ul-Islam and Haider, 2020) that reported similar effects on sperm count, motility, and morphology after the administration of CoQ10 at the dose of 100 mg twice daily for 6 months and 200 mg/daily for 3 months respectively. Furthermore, our results are consistent with those of Yamasaki et al. (Yamasaki et al., 2022) showing that daily administration of 150 mg/day of CoQ10 together with other antioxidants does not increase ejaculate volume or sperm concentration but significantly improves sperm motility. The results of Alahmar et al. (Alahmar et al., 2021) also show sperm concentration and motility improving after the administration of oral CoQ10 administration (200 mg/daily for 3 months). However, our findings are not consistent with the findings of Festa et al. (Terai et al., 2020) showing an improvement in progressive sperm motility but not sperm morphology after supplementation with 50 mg CoQ10 twice daily for 12 consecutive weeks. They are also not consistent with the findings of Stenqvist et al. (2018) showing no improvement in any of the variables of conventional semen analysis after CoQ10 supplementation at 10 mg twice daily for 3 and 6 consecutive months. This disparity may be due to the low doses of CoQ10 administered in the older studies. (Terai et al., 2020; Stenqvist et al., 2018). It is likely that a higher dose of CoQ10 is required to achieve a beneficial effect on semen quality.

The male reproductive function is regulated by the hypothalamic-pituitary-testicular (HPT) axis, which involves the pulsatile release of gonadotropin-releasing hormone. These hormones stimulate the anterior pituitary gland to release LH and FSH. LH activated the Leydig cells to produce testosterone, while FSH promote spermatogenesis by activating the Sertoli cells. (Sengupta et al., 2021). The HPT axis operates through a negative feedback loop, wherein optimal levels of testosterone suppresses LH and FSH secretion. Furthermore, inhibin B plays a crucial role in the negative regulation of FSH secretion, (Ajayi and Akhigbe, 2020) establishing an inverse relationship between inhibin B and FSH, and a positive relationship between inhibin B and sperm count (Meachem et al., 2001). Optimal levels of testosterone are also crucial for spermatogenesis (Ajayi and Akhigbe, 2020). Therefore, our results, indicating that CoQ10 supplementation considerably elevates serum testosterone and inhibin B levels while decreasing LH and FSH levels suggest an improvement in semen quality, particularly in sperm count. The improvement is likely due to the ability of CoQ10 to promote testosterone production, accompanied by an increase in inhibin B levels, which exerts a negative feedback effect on LH and FSH release. This demonstrates that CoQ10 enhances sperm count, at least in part, through a testosterone-dependent mechanism.

Apart from the hormonal mechanism through which CoQ10 may exert its effect, it also exerts antioxidant activities. Although a meta-analysis on the antioxidant activities of CoQ10 could not be conducted due to limited data in the RCTs, Safarinejad et al. (2012) demonstrate that the oral intake of 200 mg/day of CoQ10 for 26 weeks substantially increases seminal plasma catalase and superoxide dismutase (SOD) activities by 35.7% and 51.1% respectively. This is in concordance with the recent findings of Alahmar et al. (Alahmar et al., 2021) reporting that oral administration of CoQ10 at 200 mg/day for 3 months markedly improves seminal fluid total antioxidant capacity (TAC) and glutathione peroxidase (GPx) activity, while reducing ROS levels and sperm DNA fragmentation (SDF). Additionally, the study of Alahmar and Singh (Alahmar and Singh, 2022) show that oral CoQ10 at 200 mg daily for 3 months significantly increases seminal plasma TAC and catalase activity, and reduced SDF. Considering that oxidative stress can impair the HPT axis, testicular steroidogenesis, and spermatogenesis, the ability of CoQ10 to degrade ROS and increase the antioxidant status in seminal fluid explains its effect on semen quality (especially sperm count, progressive motility, and normal morphology) and testosterone production.

Of note, CoQ10 is also referred to as ubiquinol, and acts as the electron carrier in the electron transport chain complexes I, II and IV. (Fernández-Del-Río and Clarke, 2021; Turunen et al., 2004; Dallner and Sindelar, 2000). This indicates that there is a near constant biosynthesis of this molecule in cells throughout the body (Dallner and Sindelar, 2000; Thelin et al., 1992). Ubiquinol has a short half-life in the testis, 50 h (Thelin et al., 1992). Sperm cells, which are transcriptionally quiescent, cannot produce ubiquinol. Sperm cells uptake ubiquinol and ubiquinol precursors from their environment, which are converted to ubiquinol by enzymatic changes in the sperm cytoplasm or mitochondrial inner membranes (Hughes et al., 2023). The purpose of ubiquinol allocation is hypothetically in the electron transport chain and an excess would act as antioxidents in the sperm cytoplasm and membrane. In short, the demand for ubiquinol/CoQ10 in sperm cells is high throughout their lifespan, and increased levels of circulating CoQ10 or its precursors will support numerous biological functions in the testis, sperm cells and throughout the body. Hence, CoQ10 supplementation may contribute to the bioavailable ubiquinol in sperm and other testicular cells, facilitating several biological processes in the testis. This may explain the increased testosterone level in association with enhanced sperm count in CoQ10-treated men and indicates an overall improvement of testicular function (testosterone biosynthesis and spermatogenesis). The observed increased sperm motility in CoQ10-treated men also highlights the antioxidant role of CoQ10.

**FIGURE 13:**
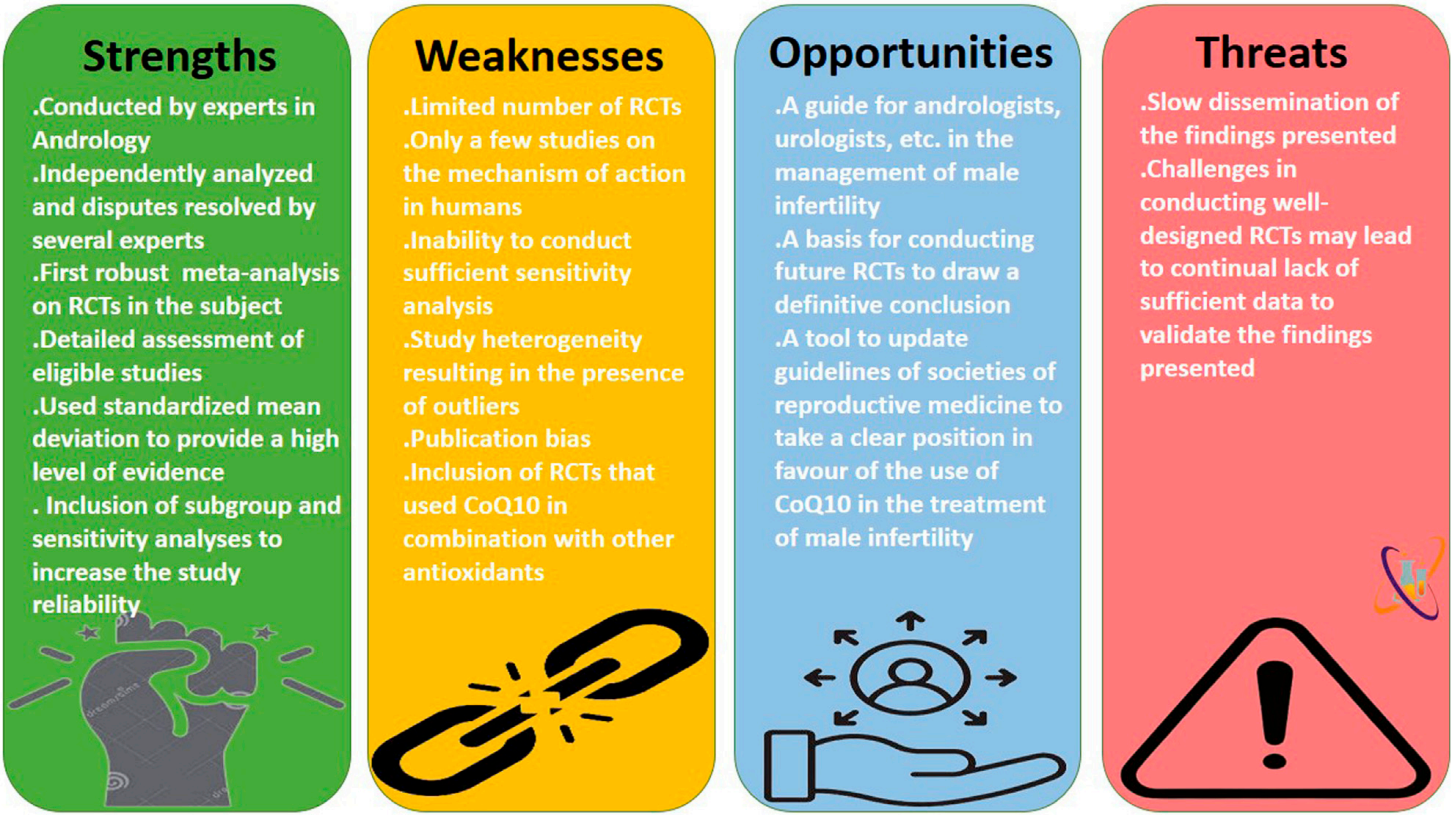
The strengths, weaknesses, opportunities, and threats (SWOT) analysis of the present meta-analysis.

This meta-analysis demonstrates the positive impact of CoQ10 on semen quality and testosterone levels using data from RCTs. However, there are some limitations. The shortage of data from the RCTs evaluating the effect of CoQ10 on semen quality reduced our pooled sample size. More so, the inclusion of the same participants at two different time-point assessments as separate arms in a meta-analysis may introduce statistical dependency, inflate sample size, and violate assumptions of independence. Also, the significant heterogeneity observed in some of the analyses culminated in the presence of outliers in the data that could not be adjusted or controlled. In addition, there were only few RCTs addressing the mechanisms of action of CoQ10 on semen quality, apart from hormonal mechanism. This limitation hampered our investigation into the antioxidant effects of CoQ10 in seminal fluid, although some conclusions could be drawn from the few available clinical data. Despite these limitations, this is the first robust meta-analysis to examine the effects of CoQ10 on semen quality and serum testosterone levels using data from rigorously conducted RCTs. Although the study of Vishvkarma et al. (2020) seems to be the first meta-analysis of RCTs on the impact of CoQ10 on semen quality, their study was limited by several factors. The study included just three studies and failed to assess the risk of bias and certainty of evidence. In addition, the effect of CoQ10 on male sex hormones, as a mechanism of action, was not reported, leaving a gap in comprehension regarding the broader implications for reproductive toxicity. Thus, the present study is a robust meta-analysis consisting 13 studies from eight published articles. Also, this study conducted a thorough assessment of the risk of bias and certainty of evidence of the included study to identify the potential source of bias. More so, sensitivity analyses were conducted by eliminating the weightiest study, studies with at high risk, and studies with low confidence of evidence to know if our findings are significantly influenced by a particular study or subset of studies, to explore the sources of heterogeneity among studies, and to provides insights into which factors are most critical, guiding future studies and helping refine research questions. In addition, subgroup analyses were conducted to explore the impact of CoQ10 as a single and combined therapy and at different treatment length (≤3 months and >3 months). Finally, the current study investigated the impact of CoQ10 on semen quality as well as serum testosterone, LH, FSH, and inhibin B, as a potential mechanism of action. Nonetheless, it is advisable to conduct further well-designed RCTs with sufficiently large samples in order to arrive at results that are more conclusive. The Strengths, Weaknesses, Opportunities, and Threats (SWOT) analysis of the current study is shown in Figure 13.

In conclusion, this meta-analysis analyzed the largest possible sample size to evaluate the effect of CoQ10 supplementation on semen quality and serum testosterone levels in RCTs. Its results suggest that CoQ10 administered alone or in combination with other antioxidants, improves sperm count, total and progressive sperm motility, and normal sperm morphology. This improvement is associated with an increase in serum testosterone and inhibin B levels, as well as a decrease in serum LH and FSH levels. Our meta-analysis aims to inform and potentially update reproductive medicine guidelines, by advocating the inclusion of CoQ10 in the management of male infertility. However, additional well-designed RCTs with sufficiently large numbers of subjects are recommended to draw definite conclusions, achieve a standardized dose/formulation, and validate whether or not the effect of CoQ10 on semen quality is testosterone-dependent.

## Data Availability

The original contributions presented in the study are included in the article/Supplementary Material, further inquiries can be directed to the corresponding author.
