# Supplementary material for: Does coenzyme Q10 improve semen quality and circulating testosterone level? a systematic review and meta-analysis of randomized controlled trials
Source: Front Pharmacol. 2025 Jan 3;15:1497930. doi: 10.3389/fphar.2024.1497930 (PMC11739123; doi:10.3389/fphar.2024.1497930)
Supplement: Supplementary file 1 [file DataSheet1.docx]

Supplementary T1: Assessment of risk of bias using Cochrane’s risk of bias tool^36^

| **Bias domain** | **Source of bias** | **Support for judgment** | **Authors’ judgment (assessed as low, unclear or high risk of bias)** |
| --- | --- | --- | --- |
| **Selection bias** | Random sequence generation | Describe the method used to generate the allocation sequence in sufficient detail to allow an assessment of whether it should produce comparable groups | Selection bias (biased allocation to interventions) due to inadequate generation of a randomised sequence |
|  | Allocation concealment | Describe the method used to conceal the allocation sequence in sufficient detail to determine whether intervention allocations could have been foreseen before or during enrolment | Selection bias (biased allocation to interventions) due to inadequate concealment of allocations before assignment |
| **Performance bias** | Blinding of participants and personnel | Describe all measures used, if any, to blind trial participants and researchers from knowledge of which intervention a participant received. Provide any information relating to whether the intended blinding was effective | Performance bias due to knowledge of the allocated interventions by participants and personnel during the study |
| **Detection bias** | Blinding of outcome assessment | Describe all measures used, if any, to blind outcome assessment from knowledge of which intervention a participant received. Provide any information relating to whether the intended blinding was effective | Detection bias due to knowledge of the allocated interventions by outcome assessment |
| **Attrition bias** | Incomplete outcome data | Describe the completeness of outcome data for each main outcome, including attrition and exclusions from the analysis. State whether attrition and exclusions were reported, the numbers in each intervention group (compared with total randomised participants), reasons for attrition or exclusions where reported, and any reinclusions in analyses for the review | Attrition bias due to amount, nature, or handling of incomplete outcome data |
| **Reporting bias** | Selective reporting | State how selective outcome reporting was examined and what was found | Reporting bias due to selective outcome reporting |
| **Other bias** | Anything else, ideally prespecified | State any important concerns about bias not covered in the other domains in the tool | Bias due to problems not covered elsewhere |

Supplementary T2: Assessment of certainty of evidence^37^

| **Initial confidence by key features of the study*** | **Factors decreasing confidence** | **Factors increasing confidence** | **Final confidence in the body of evidence** |
| --- | --- | --- | --- |
| High (++++), 4 features | 1. Risk of bias 2. Unexplained inconsistency 3. Indirectness 4. Imprecision 5. Publication bias | 1. Large magnitude effect 2. Dose response 3. Residual confounding 4. Studies report an effect and residual confounding is toward null 5. Studies report an effect and residual confounding is away from null 6. Consistency 7. Across animal models or species 8. Across dissimilar populations 9. Across study design types 10. Others: particularly rare outcomes | High (++++) |
| Moderate (+++), 3 features |  |  | Moderate (+++) |
| Low (++), 2 features |  |  | Low (++) |
| Very low (+), ≤ 1 feature |  |  | Very low (+) |
| *The features initially considered are: 1. Controlled exposure 2. Exposure prior to outcome 3. Individual outcome data 4. Comparison group used | | | |

1.
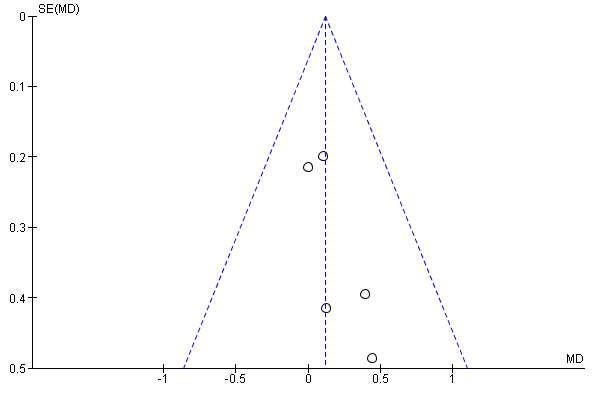
 B.
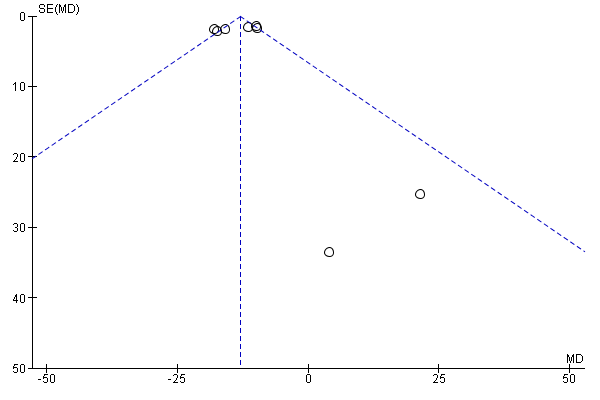
 C.
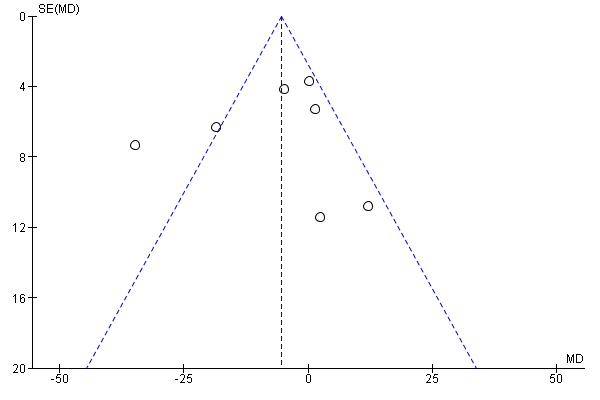


D.
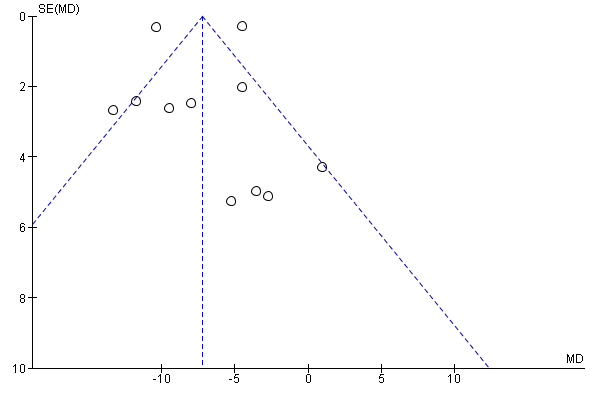
 E.
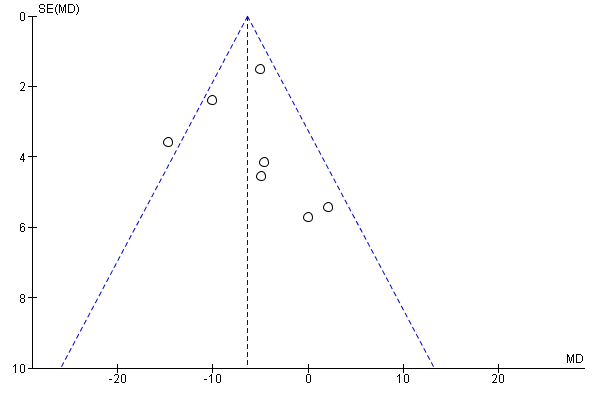
F.
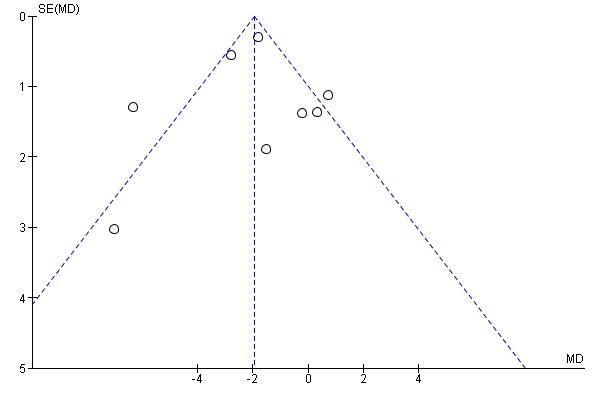


G.
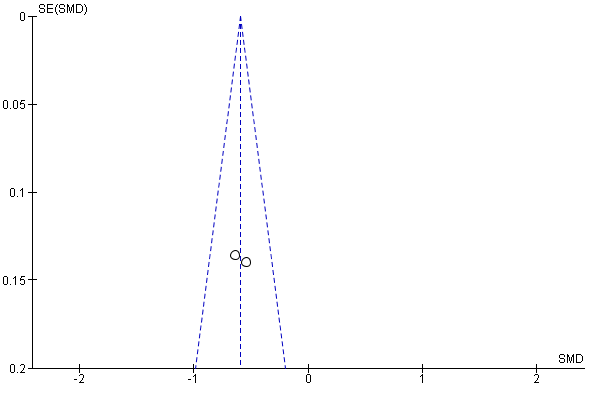
 H.
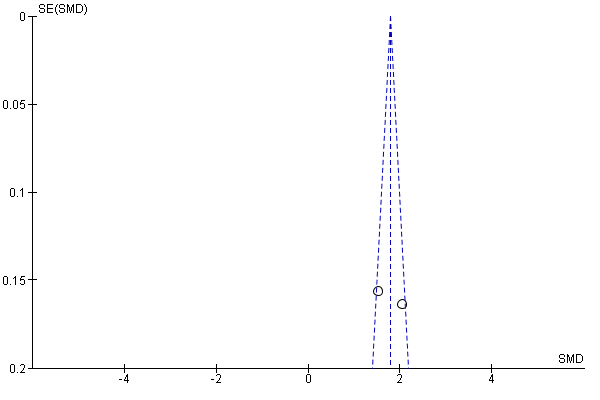
I.
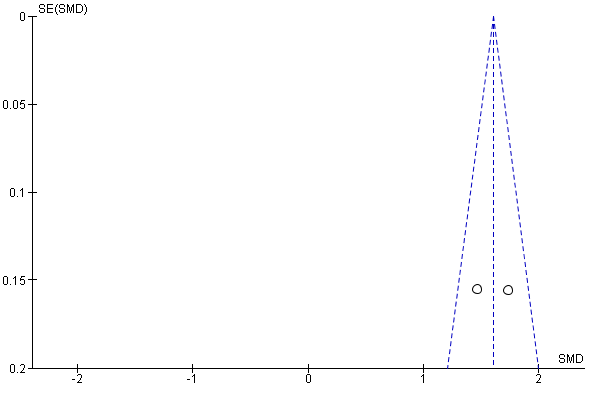


J.
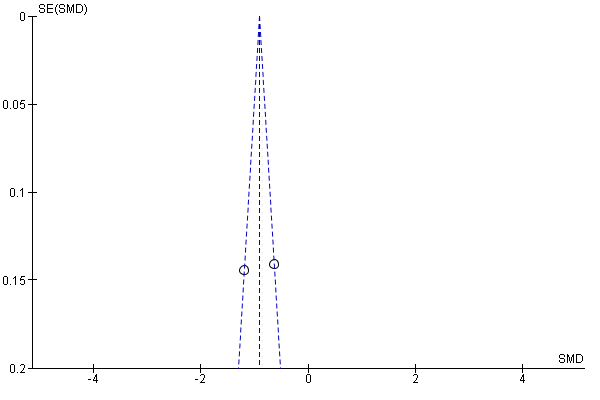


Supplementary Figure 1: Funnel plot showing the publication bias for the studies used on the efffect of CoQ10 supplementation on ejaculate volume (A), sperm count (B), sperm concentration (C), total motility (D), progressive motility (E), normal morphology (F), and serum levels of testosterone (G), luteinizing hormone, LH (H), follicle-stimulating hormone, FSH (I), and inhibin B (J)
